# Data-driven classification of prediabetes using cardiometabolic biomarkers: Data from National Health and Nutrition Examination Survey 2007–2016

**DOI:** 10.3389/fendo.2022.937942

**Published:** 2022-08-22

**Authors:** Yan Jiang, Jinying Xia, Caiyan Che, Yongning Wei

**Affiliations:** ^1^ Medical Department, Hwa Mei Hospital, University of Chinese Academy of Sciences, Ningbo, China; ^2^ Department of Endocrinology, Hwa Mei Hospital, University of Chinese Academy of Sciences, Ningbo, China; ^3^ Department of Hepatic Neoplasms, Hwa Mei Hospital, University of Chinese Academy of Sciences, Ningbo, China

**Keywords:** prediabetes, hypertension, estimated glomerular filtration rate, consensus clustering analysis, albumin to creatinine ratio

## Abstract

**Background:**

Cluster analyses have proposed different prediabetes phenotypes using glycemic parameters, body fat distribution, liver fat content, and insulin sensitivity. We aimed at classifying the subjects with prediabetes using cluster analysis and exploring the associations between prediabetes clusters with hypertension and kidney function.

**Methods:**

Patients with prediabetes in the National Health and Nutrition Examination Survey (NHANES) underwent comprehensive phenotyping and physical and laboratory variable assessment. We identified six clusters using consensus clustering analysis based on the measurements representing the body fat, glycemic status, pancreatic islet function, blood lipids, and liver function. Differences in the characteristics and prevalence of hypertension, decreased estimated glomerular filtration rate (eGFR), and increased albumin-to-creatinine ratio (ACR) were compared between clusters.

**Results:**

A total of 4,385 subjects with prediabetes were classified into six clusters of distinctive patterns by manifesting higher or lower levels of certain metabolic parameters in each cluster. Subjects with prediabetes in cluster 1 had the lowest prevalence of hypertension, decreased eGFR, and increased ACR, whereas these were much higher in cluster 5 and cluster 6. Except for cluster 3, all the other clusters had significantly increased odds ratio (OR) of hypertension as compared with cluster 1. Compared with cluster 1, all the other clusters presented significantly increased ORs of decreased eGFR. There were also significantly higher ORs of increased ACR for cluster 5 (OR 1.95, 95% confidence interval [CI] 1.09–3.51) and cluster 6 (OR 2.02, 95%CI = 1.15–3.52) compared with cluster 1.

**Conclusion:**

We stratified subjects with prediabetes into six subgroups with different characteristics. With further development and validation, such approaches might guide early intervention on the risk factors for the subjects with prediabetes who would benefit most.

## Introduction

Due to population aging, urbanization, and abrupt transition of lifestyles, the prevalence of type 2 diabetes mellitus (T2DM) is rapidly rising at tremendous speed globally. Prediabetes (intermediate hyperglycemia) is defined by fasting plasma glucose (FPG), 2-h postprandial glucose (PG), and HbA1c that are higher than normal but lower than the diabetes thresholds (1). Almost one-third of the U.S. population has prediabetes defined using FPG, 2-h PG, and HbA1c (2). Reports estimate that more than 470 million people will have prediabetes by 2030 (3).

Prediabetes is a high-risk state for diabetes development. Compared with normoglycemia, prediabetes is associated with an increased risk of all-cause mortality and cardiovascular disease (CVD) (4). The Finnish Diabetes Prevention Study proved that T2DM could be prevented by changes in lifestyle among high-risk subjects (5). The Da Qing Diabetes Prevention Outcome Study also provided strong evidence that a combination of diet and exercise intervention could halt the progression toward T2DM and reduce the incidence of CVD events in patients with prediabetes (6). However, the counterargument is that describing people with an increased risk of T2DM as having prediabetes creates more problems than benefits in terms of prevention and treatment, resulting in unnecessary medical intervention and an unsustainable burden on healthcare systems (7).

Recently, many studies that used data-driven algorithms demonstrated that T2DM was heterogeneous in its clinical features, pathogenesis, and complications (8, 9). Their findings indicated that individuals with prediabetes might also differ in metabolic features. Wagner et al. used the data-driven cluster analysis with the phenotyping variables derived from oral glucose tolerance tests, MRI-measured body fat distribution, liver fat content, and genetic risk to classify the patients with prediabetes into six clusters with different metabolic features and disease risks (10). However, since the subjects in Wagner’s study were from Europe, their findings might only be applicable to populations of European descent (10). The classification of prediabetes also needed to be tested by using other clustering strategies, changing the clustering variables, and conducting among other racial/ethnic participants with prediabetes. In the present study, we aimed to examine whether typic metabolic parameters endorsed the prediabetes clusters. Then, we postulated that specific cluster-based subphenotypes of prediabetes differently correlated with hypertension and impaired kidney function; therefore, targeted risk factor interventions were required.

## Methods

### Study population

The National Health and Nutrition Examination Survey (NHANES) was an ongoing cross-sectional nationally representative survey of the U.S. civilian population conducted by the National Center for Health Statistics (NCHS) of the Centers for Disease Control and Prevention (11). The questionnaire data, physical examination data, and biospecimens from participants were collected. Details of the study design, protocols of data collection, and datasets were publicly available (http://www.cdc.gov/nchs/nhanes.htm). The physical examinations and laboratory tests in NHANES took place in a mobile examination center using standardized protocols and calibrated equipment, and details on the data collection were described on the website (https://wwwn.cdc.gov/nchs/nhanes/analyticguidelines.aspx).

The present study analyzed data including 50,588 participants from five consecutive survey cycles (NHANES 2007–2016). The subject selection is shown in **Supplementary Figure 1**. Participants were excluded if they were pregnant at examination or uncertain of the pregnancy status (n = 715), aged younger than 18 (n = 19,864), and were not in prediabetes status (n = 25,328). Participants were also excluded due to missing data or outliers of cluster variables (n = 296). Finally, a total of 4,385 eligible subjects with prediabetes were included in the analysis. The NCHS Research Ethics Review Board reviewed and approved the study, and informed written consent was obtained from all participants before they took part in the study.

### Definitions

Body mass index (BMI) was calculated by weight (in kilograms) divided by the square of height (in meters). Insulin resistance was estimated by the homeostasis model assessment—insulin resistance (HOMA-IR) index: fasting insulin (µU/ml) × fasting glucose (mmol/L)/22.5. The β-cell function was estimated by the homeostasis model assessment β-cell function (HOMA-β) index: (20 × fasting insulin [µU/ml])/(fasting glucose [mmol/L] − 3.5). Prediabetes was defined according to the American Diabetes Association 2010 criteria, i.e., in participants without diabetes, FPG between 5.6 mmol/L and less than 7.0 mmol/L, or 2-h PG between 7.8 and less than 11.1 mmol/L, or HbA1c between 5.7% and less than 6.5% (12). Hypertension was defined as systolic blood pressure (SBP) ≥ 130 mmHg or diastolic blood pressure (DBP) ≥ 80 mmHg or currently taking antihypertensive medicine (13).

The kidney function was assessed by estimated glomerular filtration rate (eGFR) and albumin-to-creatinine ratio (ACR). The eGFR was calculated using the 2009 chronic kidney disease epidemiology collaboration (CKD-EPI) equation (**Supplementary Table 1**) (14). Albuminuria was assessed using ACR based on morning spot urine. Decreased eGFR was defined as eGFR level < 90 ml/min/1.73 m^2^; increased ACR was defined as ACR ≥ 30 mg/g (14).

### Covariates

Demographic characteristics, lifestyle factors, and currently healthy conditions were obtained through the survey by trained interviewers using questionnaires. Higher education level was defined as attaining more than the ninth grade. Current smoking was defined as having smoked at least 100 cigarettes in life and smoking at present. Current drinking was defined as taking at least 12 times drinks of any type of alcoholic beverage in the last 12 months. Physical activity was estimated using the form of the Global Physical Activity Questionnaire by asking questions about the intensity, duration, and frequency of physical activity. Total metabolic equivalent minutes per week were calculated as the measurement of physical activity level for the subjects. A higher level of physical activity was defined as having a higher metabolic equivalent/week than the median levels of the metabolic equivalent/week by cycles of survey. The information on currently taking prescribed medicine for treating hypertension, diabetes, and chronic kidney disease was investigated in the survey.

### Statistical analysis

The consensus clustering algorithm was used to classify the subjects with prediabetes based on 12 metabolic-related factors, including age at diagnosis, BMI, HbA1c, FPG, 2-h PG, HOMA-IR, HOMA-β, triglyceride (TG), high-density lipoprotein cholesterol (HDL-c), aspartate transaminase (AST), alanine transaminase (ALT), and glutamyl-transpeptidase (GGT). The unsupervised consensus clustering was always used for high-dimensional data (15). It was used to maximize the number of clusters while maintaining high cluster consensus. The cluster analysis first set a prespecified number of clusters *K* = 2, 3, …, 7, and then a random subset was created that included 80% of the original data records without replacement and repeated 100 times. For each random subset, we conducted *K*-means (Euclidean distance-based) algorithm and assigned each individual to one of the clusters. After 100 runs, the frequency of any pair of two individuals was calculated and clustered together under each scenario of *K*, and an *N*-by-*N* matrix of participantsˈ pairwise consensus value was constructed, where *N* is the sample size. The final cluster membership was determined by performing a hierarchical clustering algorithm using the consensus matrix as a measure of similarity. In the consensus matrix, consensus values ranging from 0 (never clustered together) to 1 (always clustered together) were marked by white to blue. The consensus matrix is ordered by the consensus clustering, which is depicted as a dendrogram atop the heatmap. The cluster memberships are marked by colored rectangles between the dendrogram and heatmap according to a legend with changing color to denote the similarity.

The optimal number of clusters was determined by reviewing the consensus matrix heatmap, cumulative distribution function (CDF) plot, and the within-cluster consensus scores. The CDF was defined over the range between 0 and 1. The CDF plot showed the area under the CDFs for each *K*, and at the number of clusters, the CDF reached an approximate maximum; thus, consensus and cluster confidence were at a maximum at this *K*. The relative change in area under the CDF curve comparing *K* and *K* − 1 was also used to determine the optimal number of clusters. The cluster consensus score was defined as the average consensus value for all pairs of individuals belonging to the same cluster. A value closer to one indicated better cluster stability.

Consensus clustering analysis was performed with a maximum *K* value of 7 using the *ConsensusClusterPlus* function (replication = 100, proportion of random subset = 0.8, Euclidean distance-based *K*-means algorithm) in the *ConsensusClusterPlus* package in R version 4.0.3 (http://www.r-project.org).

The appropriate weights and design factors were invoked in the analyses to account for the multistage probability sampling design of the survey. Demographic and metabolic characteristics of study participants were described in means (95% confidence intervals [CIs]) for continuous variables and percentages (95%CIs) for categorical variables in the subjects by clusters. The comparisons of metabolic-related factors between clusters were using Tukey’s test. After adjustment for potential confounders, the weighted logistic regression model was performed to evaluate the association of prediabetes clusters with hypertension, decreased eGFR, and increased ACR. The *p* < 0.05 was considered statistically significant. All the statistical analyses were conducted using the *survey* package in R version 4.0.3 (http://www.r-project.org).

## Results

We included 4,385 subjects with prediabetes who had no missing and outlier data over the 12 metabolic-related factors. The mean age of our study population was 50.6 years, 45.5% of the subjects were women, and 68.6% of the subjects were non-Hispanic white. The overall mean eGFR was 103.6 ml/min per 1.73 m^2^ (95%CI = 102.88, 104.40), and the overall ACR was 19.01 mg/g (95%CI = 15.70, 22.32 mg/g).

**Figure 1:**
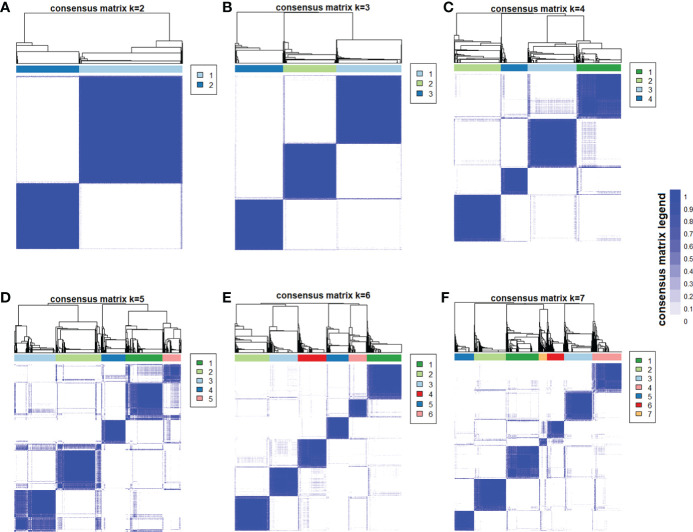
Consensus matrix heatmaps using metabolic-related factors. The consensus matrix heat maps of *K* = 2 to *K* = 7 using 12 metabolic-related factors, including age, body mass index, HbA1c, fasting glucose, 2-h postprandial glucose, homeostasis model assessment—insulin resistance, homeostasis model assessment-β, triglyceride, high-density lipoprotein cholesterol, aspartate transaminase, alanine transaminase, and glutamyl-transpeptidase (n = 4,385). The blue color represents perfect consensus where two individuals always group together, the white color represents perfect consensus where two individuals always group separately, and the blue color scales in between represent ambiguous consensus where two individuals are grouped together in some runs but separately in others. **(A)** *K* = 2. **(B)** *K* = 3. **(C)** *K* = 4. **(D)** *K* = 5. **(E)** *K* = 6. **(F)** *K* = 7.

**Figure 2:**
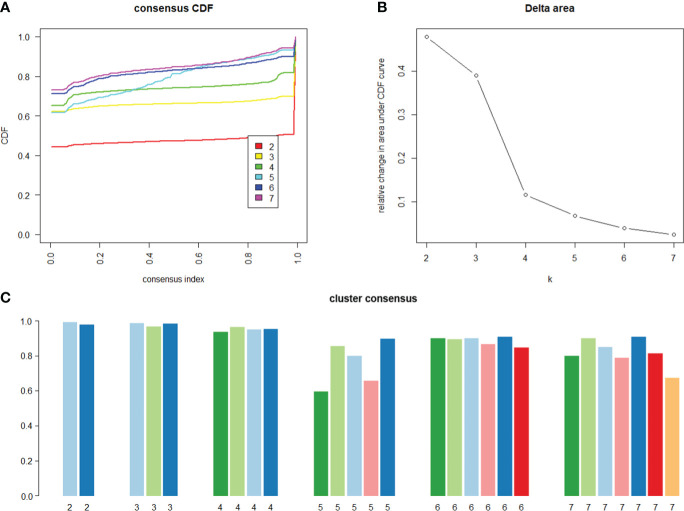
Consensus cumulative distribution function and cluster consensus score to determine what number of clusters. **(A)** The cumulative distribution functions (CDF) of the consensus matrix for each *K* (indicated by colors), estimated by a histogram of 100 bins. The CDF reaches an approximate maximum; thus, consensus and cluster confidence are at a maximum at this *K* **(B)** The relative change in area under the CDF curve comparing *K* and *K* − 1. For *K* = 2, there is no *K* − 1, so the total area under the curve rather than the relative increase is plotted. The relative increases in consensus are used to determine *K* at which there is an appreciable increase. **(C)** The bar plot represents the mean consensus score for different numbers of clusters (*K* ranges from two to seven) on the basis of 100 repeated re-samplings of 80% of the 4,385 prediabetic participants. Cluster is indicated by color following the same color scheme as the cluster matrices and tracking plots. The bars are grouped by *K*, which is marked on the horizontal axis. High values indicate that a cluster has high stability, and low values indicate a cluster has low stability. For *K* = 6, the mean consensus score was 0.90 for cluster 1, 0.89 for cluster 2, 0.90 for cluster 3, 0.86 for cluster 4, 0.91 for cluster 5, and 0.85 for cluster 6.

With the use of the 12 metabolic-related factors, the consensus clustering algorithm identified six clusters that best represent the data pattern of prediabetes subjects. By visualizing the matrix heatmaps of the pairwise consensus for each cluster size in **Figure 1**, the CDFs in **Figure 2A**, and the proportion increase of the area under the CDFs in **Figure 2B**, *K* = 6 was the largest number of clusters that was reasonably considered. For *K* = 6, the mean consensus score was 0.90 for cluster 1, 0.89 for cluster 2, 0.90 for cluster 3, 0.86 for cluster 4, 0.91 for cluster 5, and 0.85 for cluster 6, with a larger value indicating better stability of cluster membership (**Figure 2C**). A sensitivity analysis was also conducted among the subjects with prediabetes currently not taking lipid-lowering medication and without a history of major diseases using the unsupervised consensus cluster algorithm. **Supplementary Figures 2, 3** also show that the classification of prediabetes into six clusters is also the optimum. The percentages of abnormal glycemic parameters (including HbA1c ≥ 5.7%, FPG ≥ 5.6 mmol/L, and 2-h PG ≥ 7.8 mmol/L) were significantly different between clusters (**Supplementary Figure 4**). Subjects with prediabetes in cluster 1 were constituted mostly by FPG ≥ 5.6 mmol/L. Subjects with prediabetes in cluster 3 had the highest percentage of HbA1c ≥ 5.7% but the lowest percentage of fasting glucose ≥ 5.6 mmol/L. The percentages of abnormal glycemic parameters were similar between cluster 4 and cluster 6.

**Figure 3:**
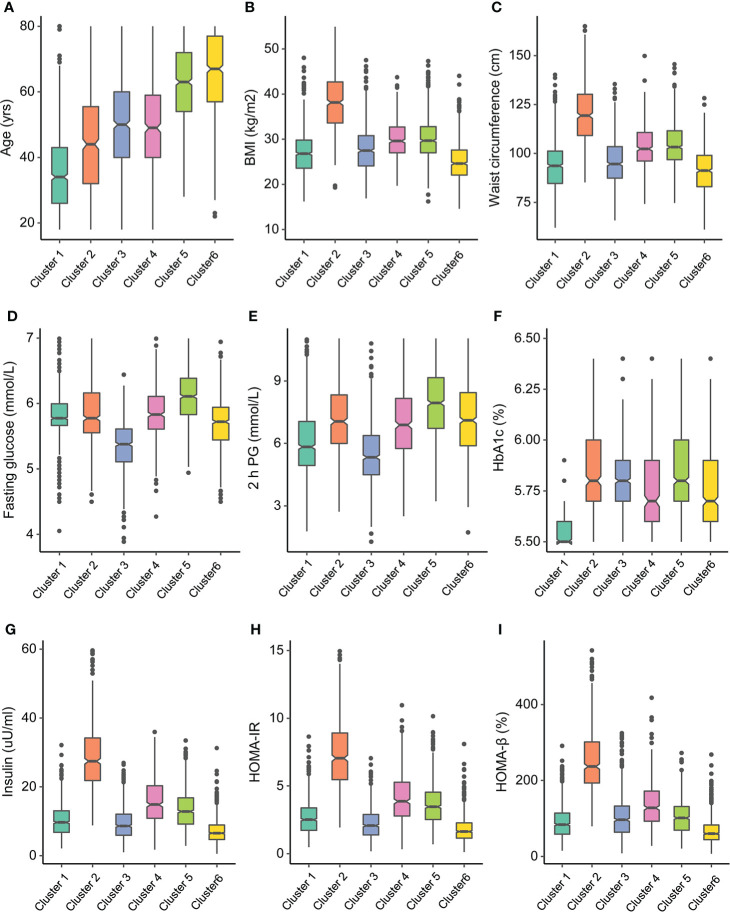
Participant cluster characteristics. Distributions of **(A)** age at diagnosis, **(B)** body mass index (BMI), **(C)** waist circumference, **(D)** fasting glucose, **(E)** 2-h postprandial glucose, **(F)** HbA1c, **(G)** insulin, **(H)** homeostasis model assessment—insulin resistance (HOMA-IR), and **(I)** homeostasis model assessment-β (HOMA-β) at baseline for each cluster.

**Figure 4:**
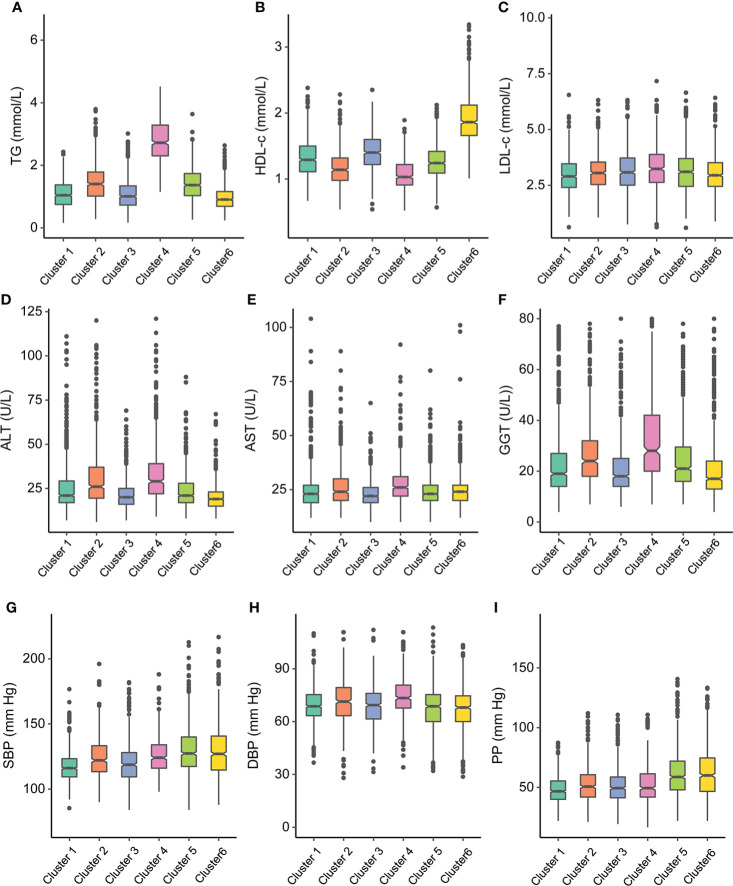
Participant cluster characteristics. Distributions of **(A)** triglyceride (TG), **(B)** high-density lipoprotein cholesterol (HDL-c), **(C)** low-density lipoprotein cholesterol (LDL-c), **(D)** alanine transaminase (ALT), **(E)** aspartate transaminase (AST), **(F)** glutamyl-transpeptidase (GGT) at baseline for each cluster, **(G)** systolic blood pressure (SBP), **(H)** diastolic blood pressure (DBP), and **(I)** pulse pressure (PP) at baseline for each cluster.

The characteristics of the clusters are listed in **Table 1**. As shown in **Figures 3**, **4**, each cluster had distinctive key features. Cluster 1 represented the most frequent cluster in this population (23.2%) and was characterized by the lowest age at diagnosis and the lowest HbA1c and ACR levels. They also possessed a lower level of BMI, waist circumference (WC), 2-h PG, insulin, HOMA-IR, HOMA-β, TG, and low-density lipoprotein cholesterol (LDL-c). Cluster 2 comprised 12.8% of clustered subjects. These individuals had the highest BMI and WC, as well as the highest levels of insulin, HOMA-IR, HOMA-β, TG, and HDL-c. Cluster 3 constituted 15.0% of the subjects. This group was characterized by the lowest levels of FPG and 2-h PG, as well as the lowest levels of ALT, AST, and GGT. They also presented lower levels of BMI, WC, insulin, HOMA-IR, and HOMA-β. Cluster 4 included 480 (12.9%) subjects and was marked by the lowest HDL-c level and the highest levels of TG, LDL-c, ALT, AST, and GGT. Cluster 5 comprised 18.7% of clustered subjects. This group had the highest level of FPG and 2-h PG. Cluster 6 comprised 17.4% of clustered subjects. They had the oldest age and the lowest levels of insulin, HOMA-IR, HOMA-β, TG, and eGFR but the highest level of ACR.

**Table 1:** The characteristics of prediabetes subjects by clusters.

	Cluster 1	Cluster 2	Cluster 3	Cluster 4	Cluster 5	Cluster 6
Number, n (%)	916 (23.2)	571 (12.8)	746 (15.0)	480 (12.9)	899 (18.7)	773 (17.4)
Female (%)	0.31 (0.27, 0.34)	0.52 (0.48, 0.57)	0.51 (0.46, 0.55)	0.32 (0.27, 0.37)	0.44 (0.40, 0.48)	0.67 (0.62, 0.72)
Age, year	35.96 (34.66, 37.26)	43.46 (41.59, 45.33)	50.34 (49.12, 51.57)	48.90 (47.53, 50.28)	61.34 (60.15, 62.53)	63.65 (62.46, 64.83)
Race/ethnicity (%)						
Mexican American	0.13 (0.10, 0.16)	0.12 (0.09, 0.16)	0.09 (0.07, 0.12)	0.10 (0.06, 0.13)	0.07 (0.05, 0.09)	0.03 (0.02, 0.04)
Other Hispanic	0.07 (0.05, 0.09)	0.06 (0.04, 0.08)	0.06 (0.04, 0.08)	0.05 (0.03, 0.07)	0.05 (0.04, 0.07)	0.03 (0.02, 0.04)
Non-Hispanic white	0.65 (0.60, 0.70)	0.62 (0.56, 0.68)	0.56 (0.50, 0.62)	0.76 (0.71, 0.81)	0.74 (0.69, 0.78)	0.78 (0.74, 0.82)
Non-Hispanic black	0.06 (0.04, 0.07)	0.15 (0.11, 0.18)	0.23 (0.18, 0.27)	0.03 (0.01, 0.04)	0.07 (0.06, 0.09)	0.08 (0.06, 0.10)
Others	0.09 (0.07, 0.11)	0.05 (0.03, 0.07)	0.06 (0.04, 0.08)	0.07 (0.04, 0.09)	0.07 (0.05, 0.10)	0.08 (0.05, 0.10)
High level of education (%)	0.59 (0.54, 0.64)	0.61 (0.56, 0.66)	0.53 (0.47, 0.60)	0.57 (0.49, 0.66)	0.54 (0.49, 0.59)	0.67 (0.62, 0.71)
Currently smoking (%)	0.41 (0.36, 0.46)	0.41 (0.35, 0.47)	0.51 (0.45, 0.57)	0.55 (0.50, 0.60)	0.53 (0.49, 0.57)	0.43 (0.39, 0.48)
Currently drinking (%)	0.80 (0.77, 0.84)	0.67 (0.61, 0.73)	0.71 (0.65, 0.77)	0.83 (0.79, 0.88)	0.74 (0.70, 0.79)	0.77 (0.71, 0.82)
High level of activity (%)	0.63 (0.59, 0.67)	0.43 (0.37, 0.49)	0.57 (0.51, 0.63)	0.53 (0.47, 0.58)	0.48 (0.43, 0.53)	0.52 (0.47, 0.56)
Taking antihypertensive agents (%)	0.11 (0.08, 0.14)	0.37 (0.31, 0.43)	0.26 (0.21, 0.31)	0.30 (0.25, 0.36)	0.47 (0.42, 0.52)	0.38 (0.33, 0.43)
Taking lipid-lowering agents (%)	0.07 (0.04, 0.15)	0.17 (0.13, 0.36)	0.22 (0.17, 0.45)	0.22 (0.17, 0.47)	0.37 (0.32, 0.74)	0.31 (0.27, 0.64)
BMI, kg/m^2^	27.39 (26.96, 27.83)	39.93 (39.14, 40.71)	27.84 (27.31, 28.37)	30.12 (29.45, 30.79)	30.57 (29.99, 31.14)	25.17 (24.73, 25.62)
WC, cm	94.95 (93.84, 96.06)	122.86 (121.33, 124.39)	96.58 (95.2, 97.95)	103.96 (102.25, 105.68)	106.39 (105.01, 107.77)	91.78 (90.52, 93.03)
FPG, mmol/L	5.81 (5.78, 5.84)	5.89 (5.83, 5.95)	5.38 (5.34, 5.43)	5.85 (5.79, 5.91)	6.13 (6.09, 6.17)	5.72 (5.67, 5.76)
2-hour PG, mmol/L	6.03 (5.90, 6.16)	7.14 (6.95, 7.34)	5.34 (5.19, 5.49)	7.04 (6.83, 7.24)	7.91 (7.76, 8.05)	6.99 (6.83, 7.14)
HbA1c, %	5.20 (5.18, 5.23)	5.64 (5.61, 5.67)	5.79 (5.77, 5.81)	5.51 (5.46, 5.56)	5.73 (5.70, 5.76)	5.55 (5.53, 5.58)
Insulin,	10.14 (9.69, 10.58)	28.93 (28.12, 29.75)	9.19 (8.68, 9.69)	15.17 (14.22, 16.12)	13.36 (12.84, 13.88)	7.02 (6.69, 7.34)
HOMA-IR	2.63 (2.51, 2.75)	7.64 (7.37, 7.91)	2.21 (2.08, 2.35)	3.97 (3.71, 4.23)	3.65 (3.50, 3.81)	1.79 (1.71, 1.88)
HOMA-β	88.95 (85.11, 92.79)	246.39 (240.05, 252.74)	100.87 (96.05, 105.7)	130.64 (122.82, 138.47)	103.03 (99.41, 106.66)	64.62 (61.56, 67.69)
HDL-c, mmol/L	1.30 (1.27, 1.33)	1.12 (1.10, 1.15)	1.40 (1.37, 1.43)	1.06 (1.03, 1.09)	1.27 (1.24, 1.29)	1.94 (1.9, 1.98)
TG, mmol/L	1.08 (1.04, 1.12)	1.52 (1.45, 1.59)	1.1 (1.06, 1.15)	2.83 (2.76, 2.91)	1.41 (1.37, 1.46)	0.95 (0.91, 0.99)
LDL-c, mmol/L	2.99 (2.94, 3.05)	3.08 (2.98, 3.17)	3.19 (3.10, 3.28)	3.34 (3.21, 3.48)	3.09 (3.00, 3.18)	3.00 (2.90, 3.10)
AST, U/L	24.07 (23.49, 24.65)	26.41 (25.5, 27.32)	23.35 (22.56, 24.15)	27.43 (26.38, 28.47)	24.55 (23.81, 25.28)	25.01 (24.36, 25.67)
ALT, U/L	25.22 (24.19, 26.24)	31.9 (30.27, 33.53)	21.81 (21.05, 22.57)	33.52 (31.30, 35.74)	24.31 (23.36, 25.27)	20.86 (20.19, 21.53)
GGT, U/L	23.80 (22.19, 25.41)	29.26 (27.22, 31.30)	21.30 (20.03, 22.56)	39.35 (34.71, 44.00)	28.59 (26.31, 30.87)	22.02 (20.61, 23.43)
SBP, mm Hg	117.89 (116.72, 119.07)	124.11 (122.44, 125.78)	119.56 (117.89, 121.24)	126.63 (124.88, 128.39)	127.51 (125.83, 129.19)	127.01 (125.38, 128.64)
DBP, mm Hg	69.65 (68.59, 70.71)	71.48 (70.03, 72.93)	68.71 (67.56, 69.87)	74.58 (73.13, 76.02)	68.27 (67.08, 69.46)	67.76 (66.37, 69.16)
PP, mm Hg	48.25 (47.23, 49.27)	52.63 (50.23, 55.04)	50.85 (49.40, 52.30)	52.06 (50.63, 53.48)	59.74 (57.66, 61.82)	58.74 (57.31, 60.17)
eGFR, ml/min/1.73 m^2^	113.55 (112.31, 114.79)	110.87 (109.04, 112.70)	106.40 (104.96, 107.84)	101.80 (100.24, 103.36)	94.07 (92.71, 95.44)	94.48 (93.02, 95.94)
ACR, mg/g	12.57 (9.17, 15.98)	19.57 (12.54, 26.60)	15.22 (11.21, 19.22)	14.66 (10.67, 18.64)	22.51 (15.19, 29.82)	29.99 (17.97, 42.01)

Data are weighted means (95% confidence interval) for continuous variables and weighted percentages (95% confidence interval) for categorical variables.

BMI, body mass index; WC, waist circumference; FPG, fasting plasma glucose; PG, postprandial glucose; HbA1c, glycated hemoglobin; HOMA-IR, homeostasis model assessment—insulin resistance; HOMA-β, homeostasis model assessment-β; HDL-c, high-density lipoprotein cholesterol; LDL-c, low-density lipoprotein cholesterol; TG, triglyceride; AST, aspartate transaminase; ALT, alanine transaminase; GGT, glutamyl-transpeptidase; SBP, systolic blood pressure; DBP, diastolic blood pressure; PP, pulse pressure; eGFR, estimated glomerular filtration rate; ACR, albumin-to-creatinine ratio.

**Supplementary Figures 5, 6** and **Supplementary Table 2** show the pairwise comparisons of the metabolic-related factors between clusters. Most differences achieved statistical significance and presented substantial differences between clusters, which were also in accordance with the key features of each cluster.

**Table 2** shows the prevalence and odds ratios (ORs) for the associations between prediabetes clusters with hypertension and impaired kidney function. Subjects with prediabetes in cluster 1 had the lowest prevalence of high SBP (0.23%, 95%CI = 0.19, 0.26%) and high DBP (0.21%, 95%CI = 0.18, 0.25%). Except for cluster 3, all the other clusters had significantly increased ORs of high SBP and hypertension compared with cluster 1. The subjects with prediabetes in cluster 2 and cluster 4 had significantly increased ORs of high DBP compared with those in cluster 1. The associations between prediabetes clusters and hypertension were not changed when defined hypertension as systolic blood pressure ≥ 140 mmHg or diastolic blood pressure ≥ 90 mmHg or currently taking antihypertensive medicine (**Supplementary Table 3**). Subjects with prediabetes in cluster 1 had the lowest prevalence of decreased eGFR (0.04%, 95%CI = 0.02, 0.09%) and increased ACR (0.05%, 95%CI = 0.03, 0.10%). Compared with cluster 1, all the other clusters presented significantly increased ORs of decreased eGFR, and cluster 6 had the largest OR of 15.11 (95%CI = 5.23, 15.63). There were significantly higher ORs of increased ACR for cluster 5 (OR = 1.95, 95%CI = 1.09, 3.51) and cluster 6 (OR = 2.02, 95%CI = 1.15, 3.52) compared with cluster 1.

**Table 2:** The association between prediabetes clusters with hypertension and kidney function.

	Cluster 1	Cluster 2	Cluster 3	Cluster 4	Cluster 5	Cluster 6
**High systolic blood pressure or taking antihypertensive medicine**						
Prevalence	0.23 (0.19, 0.26)	0.54 (0.48, 0.60)	0.39 (0.34, 0.45)	0.49 (0.42, 0.56)	0.68 (0.64, 0.73)	0.57 (0.52, 0.62)
Unadjusted OR	Reference	3.98 (2.93, 5.39)	2.21 (1.63, 3.00)	3.30 (2.39, 4.56)	7.35 (5.64, 9.58)	4.48 (3.48, 5.78)
Adjusted OR*	Reference	2.06 (1.38, 3.07)	1.37 (0.88, 2.13)	2.54 (1.57, 4.11)	3.88 (2.69, 5.61)	3.33 (2.44, 4.53)
**High diastolic blood pressure or taking antihypertensive medicine**						
Prevalence	0.21 (0.18, 0.25)	0.55 (0.50, 0.61)	0.36 (0.31, 0.41)	0.51 (0.44, 0.58)	0.58 (0.53, 0.64)	0.46 (0.40, 0.51)
Unadjusted OR	Reference	4.55 (3.32, 6.24)	2.03 (1.49, 2.76)	3.80 (2.68, 5.39)	5.14 (3.79, 6.98)	3.06 (2.25, 4.18)
Adjusted OR*	Reference	3.92 (2.44, 6.29)	1.10 (0.62, 1.95)	3.85 (2.29, 6.46)	1.72 (0.97, 3.03)	1.31 (0.66, 2.62)
**Hypertension**						
Prevalence	0.28 (0.24, 0.32)	0.61 (0.55, 0.66)	0.43 (0.38, 0.49)	0.59 (0.52, 0.65)	0.71 (0.66, 0.76)	0.59 (0.54, 0.64)
Unadjusted OR	Reference	4.02 (3.01, 5.39)	1.97 (1.49, 2.61)	3.72 (2.70, 5.12)	6.37 (4.76, 8.53)	3.76 (2.94, 4.81)
Adjusted OR*	Reference	2.70 (1.84, 3.97)	1.21 (0.78, 1.88)	3.10 (2.00, 4.80)	3.02 (2.03, 4.49)	2.57 (1.76, 3.74)
**Decreased eGFR or receiving dialysis treatment**						
Prevalence	0.04 (0.02, 0.09)	0.10 (0.07, 0.22)	0.17 (0.12, 0.35)	0.17 (0.12, 0.35)	0.42 (0.38, 0.85)	0.43 (0.38, 0.86)
Unadjusted OR	Reference	2.58 (1.51, 4.42)	4.50 (2.55, 7.93)	4.62 (2.76, 7.75)	16.59 (10.09, 27.30)	16.96 (10.20, 28.19)
Adjusted OR*	Reference	1.93 (1.06, 3.52)	3.38 (1.80, 6.33)	2.80 (1.59, 4.93)	9.04 (5.23, 15.63)	15.11 (5.23, 15.63)
**Increased ACR or receiving dialysis treatment**						
Prevalence	0.05 (0.03, 0.10)	0.09 (0.06, 0.19)	0.06 (0.04, 0.13)	0.07 (0.04, 0.14)	0.11 (0.09, 0.23)	0.11 (0.09, 0.24)
Unadjusted OR	Reference	2.01 (1.20, 3.37)	1.35 (0.80, 2.27)	1.43 (0.81, 2.53)	2.55 (1.67, 3.88)	2.65 (1.80, 3.91)
Adjusted OR*	Reference	1.35 (0.62, 2.94)	0.83 (0.36, 1.96)	1.29 (0.60, 2.77)	1.95 (1.09, 3.51)	2.02 (1.15, 3.52)
**Decreased eGFR or increased ACR or receiving dialysis treatment**						
Prevalence	0.08 (0.06, 0.11)	0.18 (0.14, 0.23)	0.21 (0.17, 0.26)	0.22 (0.17, 0.27)	0.47 (0.43, 0.52)	0.48 (0.43, 0.53)
Unadjusted OR	Reference	2.42 (1.58, 3.71)	2.92 (1.89, 4.52)	3.07 (2.10, 4.49)	9.70 (6.69, 14.06)	9.96 (6.79, 14.59)
Adjusted OR*	Reference	1.93 (1.09, 3.42)	2.17 (1.27, 3.71)	2.12 (1.32, 3.40)	6.17 (3.87, 9.84)	9.39 (5.90, 14.92)

Data are weighted prevalence rate (95% confidence interval) and weighted odds ratio (95% confidence interval). Decreased estimated glomerular filtration rate (eGFR) was defined as eGFR level < 90 ml/min/1.73 m^2^; increased albumin-to-creatinine ratio (ACR) was defined as ACR ≥ 30 mg/g. High systolic blood pressure was defined as systolic blood pressure ≥ 130 mmHg; high diastolic blood pressure was defined as diastolic blood pressure ≥ 80 mmHg.

*Adjusted model was adjusted for gender, smoking status, drinking status, education level, physical activity, taking antihypertensive medicine, and taking lipid-lowering medicine.

## Discussion

In this study, we applied the unsupervised consensus clustering algorithm and identified six clusters based on 12 metabolic-related variables. The findings of our study showed that there were distinct metabolic characteristics and different associations with hypertension and impaired kidney function between clusters. The data-driven approach used by Wagner and colleagues (10) was reproducible in the U.S. population with prediabetes; there were differences in the cluster profiles of subjects with prediabetes between our study and theirs. Even though the thresholds for defining prediabetes have been used by main international medical organizations for more than 10 years, there is still controversy surrounding the characterization of prediabetes as a distinct pathogenic condition (16). The new classification of prediabetes may indicate that prediabetes can be caused by a more complicated pathological course manifested by distinctive phenotypes in each cluster.

The clusters of prediabetes are reproducible and can help to distinguish the subjects with prediabetes into subgroups with different metabolic features. As shown in our findings, cluster 1 accounting for almost one-quarter of subjects with prediabetes was only characterized by higher FPG. They presented the lowest possibilities of having hypertension and impaired kidney function. A previous study also found that the subjects in impaired glucose tolerance defined by FPG < 6.1 mmol/L and 2-h PG 7.8 ~ 11.1 mmol/L were associated with chronic kidney disease, but not for those with impaired fasting glucose defined by FPG 6.1 ~ 7.0 mmol/L and 2-h PG < 7.8 mmol/L (17). Subjects with prediabetes in cluster 2 represented an obesity-related insulin-resistant phenotype, in which participants also had hyperinsulinemia and a higher level of HOMA-IR and HOMA-β. It might imply that there was a compensation for insulin resistance through elevated β-cell function in secreting insulin. The subjects with prediabetes in cluster 3 had the lowest levels of FPG and 2-h PG but the highest level of HbA1c than the other clusters. The subjects in cluster 3 were also significantly associated with an increased possibility of having decreased eGFR. The observation might indicate the importance of maintaining a low level of HbA1c in preventing chronic kidney disease. It should be noticed that the subjects with prediabetes in cluster 4 did not have very high blood glucose levels, but the poor liver function also prompted their impaired kidney function.

In Wagner’s study, subjects with prediabetes with a high level of visceral fat had the highest risk of chronic kidney disease. However, in our study, the subjects with prediabetes in cluster 5 characterized by the highest level of FPG and 2-h PG and in cluster 6 by the highest age presented similar and the strongest tendency of decreased eGFR, increased ACR, and hypertension. The differences between our study and theirs might be because we included age at diagnosis in the cluster analysis, while age was not used in Wagner’s study. Age was an important factor in determining the risk of chronic kidney function; therefore, the subjects with prediabetes in cluster 6 were more likely to have hypertension and poor kidney function. Age at diagnosis was also used by previous studies to classify the subphenotypes of diabetes (8). There were also other differences between Wagner’s study and ours. The subjects in Wagner’s study are the population of European descent, whereas the multiracial participants were included in the present study.

Because nearly all patients with T2DM pass through an extensive phase of prediabetes, targeting subjects with prediabetes with effective interventions can significantly alter the progression to T2DM. Nevertheless, the robust evidence from trials had demonstrated that intensive lifestyle interventions to achieve modest weight loss can yield health benefits. However, the effort to translate and implement diabetes prevention in clinical practice has lagged. There are several reasons for the difficulties. As prediabetes is asymptomatic, the prediabetic individuals are unaware of their hyperglycemia condition. The intervention can also be met with reluctance and declined if there are subtle effects. The lifestyle interventions implemented in trials are resource-intensive; even though using lifestyle modification is cost-effective (18), the cost of intervention can be expensive and complicated to implement, maintain, and reimburse (19). The novel classification of prediabetes in our study is helpful for revealing the metabolic heterogeneity, and it also suggests potential therapeutic implications. The individuals at high risk for diabetes might benefit most from the cluster-based prevention strategy, especially the people with limited healthcare and societal resources. For example, individuals in cluster 2 and cluster 4 might benefit the most from high-intensity dietary and/or lifestyle interventions aimed at weight loss and visceral fat reduction. Subjects with prediabetes in cluster 5 and cluster 6 should focus more attention on the development of hypertension and chronic kidney disease. However, it is premature to implement our clustering approach to provide definitive subphenotypes for prediabetes; future clinical trials are needed to verify such a strategy before conducting it in clinical practice.

A major strength of the present study was that the participants were recruited from a nationwide survey across the United States; a certain level of representativeness of the population could be justified. The cluster method used in our study was another strength. Different from previous similar cluster studies of prediabetes or diabetes, we used the consensus clustering approach in the present study. Unsupervised consensus clustering using discrete data elements was one approach to uncover that heterogeneity, and it determined the optimal number of clusters by objective measures. This algorithm could translate large amounts of data to clinically relevant groupings of patients with distinct clinical characteristics. Another study also used consensus clustering to categorize the patients with chronic kidney diseases into three distinct subgroups (20). However, we also acknowledged the limitations. First, there was uncertainty regarding the variables used in the cluster analysis; other biomarkers such as inflammatory factors and genetic risk score might contribute to another new subgroup. Second, there was a potential bias due to survey non-response and the absence of values for some of the anthropometric variables and biomarkers. Third, due to only 14 cases having eGFR < 60 ml/min/1.73 m^2^, we could not compare the prevalence of chronic kidney disease between different prediabetes clusters. A meta-analysis reported that prediabetes was associated with increased composite cardiovascular events, such as coronary heart disease and stroke (21). However, due to the cross-sectional nature, we could not infer the causality between the prediabetes clusters and cardiovascular diseases. Further studies with the longitudinal design were needed to explore the development of prediabetes clusters and verify the associations between clusters with chronic kidney diseases and cardiovascular diseases.

In summary, we showed that there was metabolic heterogeneity among the subjects with prediabetes defined by currently used criteria. The subphenotypes of prediabetes identified in our study present distinct metabolic characteristics and were associated with different risks of hypertension and impaired kidney function. With further development and validation, such approaches might guide early intervention on the risk factors for the subjects with prediabetes who would benefit the most.

## Data availability statement

The datasets presented in this study can be found in online repositories. The names of the repository/repositories and accession number(s) can be found below: https://wwwn.cdc.gov/nchs/nhanes/Default.aspx.

## Ethics statement

The studies involving human participants were reviewed and approved by National Center for Health Statistics Research Ethics Review Board. The patients/participants provided their written informed consent to participate in this study.

## Author contributions

YW conceived and designed the study. YJ analyzed data. YW and YJ interpreted the data. YJ drafted the manuscript. JX and CC revised it. All authors agreed to be accountable for all aspects of the work and approved the final version of the paper. YW is the guarantor of this work and, as such, has full access to all the data in the study and takes responsibility for the integrity of the data and the accuracy of the data analysis.

## Funding

This work was supported by the grant from Zhejiang Provincial Co-construction Project of Key Medical Discipline (2016-S04).

## Acknowledgments

We acknowledge the investigators and participants of the National Health and Nutrition Examination Survey.

## Conflict of interest

The authors declare that the research was conducted in the absence of any commercial or financial relationships that could be construed as a potential conflict of interest.

## Publisher’s note

All claims expressed in this article are solely those of the authors and do not necessarily represent those of their affiliated organizations, or those of the publisher, the editors and the reviewers. Any product that may be evaluated in this article, or claim that may be made by its manufacturer, is not guaranteed or endorsed by the publisher.
