# Supplementary material for: Data-driven classification of prediabetes using cardiometabolic biomarkers: Data from National Health and Nutrition Examination Survey 2007–2016
Source: Front Endocrinol (Lausanne). 2022 Aug 22;13:937942. doi: 10.3389/fendo.2022.937942 (PMC9441552; doi:10.3389/fendo.2022.937942)
Supplement: Supplementary file 1 [file DataSheet_1.docx]

296 subjects were excluded for having missing data or outliers in the cluster variables

4681 subjects with prediabetes

45,907 were excluded

- 715 being pregnant at examination or uncertain of the pregnancy status
- 19,864 with age younger than 18
- 25,328 were not in prediabetes status

50,588 subjects from National Health and Nutrition Examination Survey 2007 to 2016

4385 subjects with prediabetes were included

**Supplementary figure 1**. Participant flow diagram of the subjects


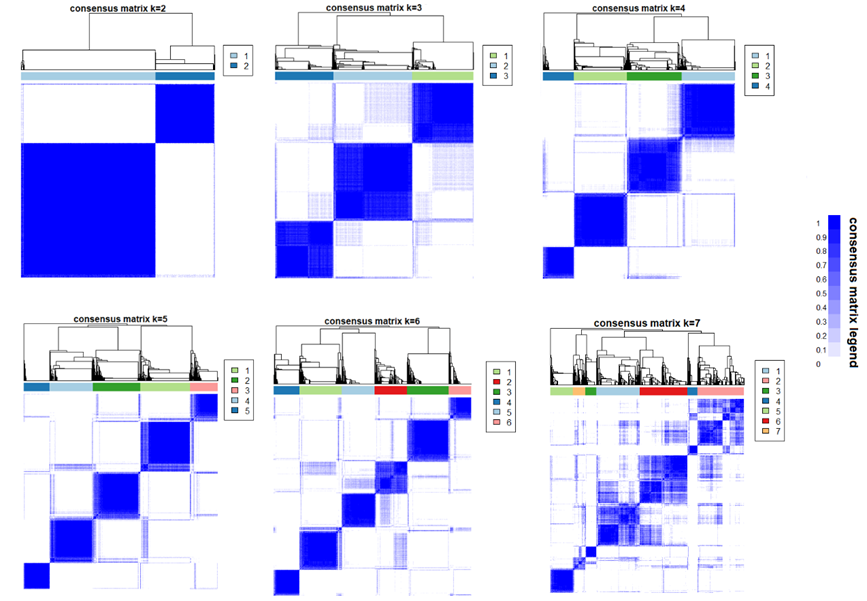


**Supplementary figure 2. Sensitivity analysis using unsupervised consensus cluster among the subjects currently not taking lipid lowering medication and without a history of major diseases.** The consensus matrix heat maps of *K*=2 to *K*=7 using 12 metabolic related factors, including age, body mass index, HbA1c, fasting glucose, 2-hour postprandial glucose, homeostasis model assessment - insulin resistance, homeostasis model assessment – β, triglyceride, high-density lipoprotein cholesterol, aspartate transaminase, alanine transaminase, glutamyl-transpeptidase.

The darkest blue color represents perfect consensus where two individuals always group together, the white color represents perfect consensus where two individuals always group separately, and the blue color scales in between represent ambiguous consensus where two individuals are grouped together in some runs but separately in others. (A) *K*=2. (B) *K*=3. (C) *K*=4. (D) *K*=5. (E) *K*=6. (F) *K*=7.


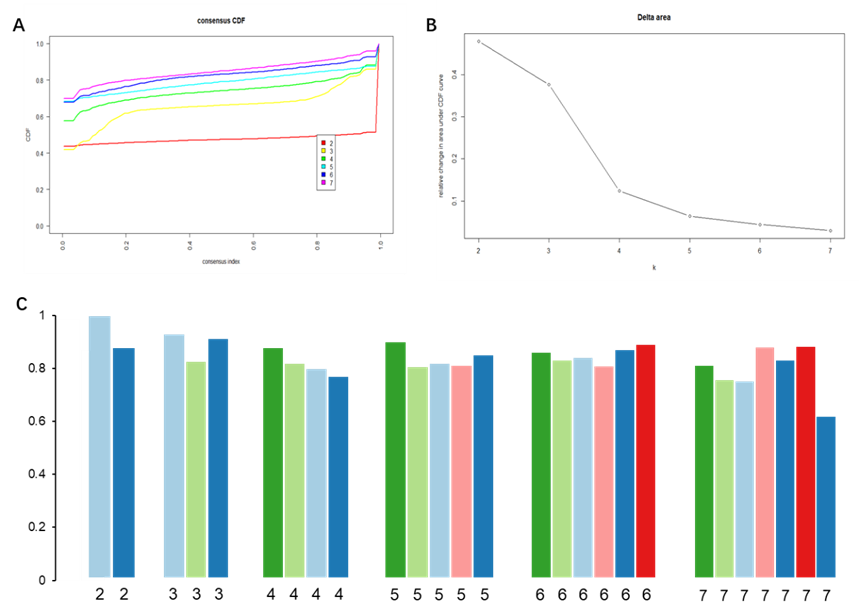


**Supplementary figure 3. Consensus cumulative distribution function and cluster consensus score to determine at what number of clusters in the sensitivity analysis conducted among the subjects currently not taking lipid lowering medication and without a history of major diseases**

The graphic in (A) shows the cumulative distribution functions (CDF) of the consensus matrix for each K (indicated by colors), estimated by a histogram of 100 bins. The CDF reaches an approximate maximum, thus consensus and cluster confidence is at a maximum at this K. The graphic in (B) shows the relative change in area under the CDF curve comparing K and K - 1. For K = 2, there is no K -1, so the total area under the curve rather than the relative increase is plotted. The relative increases in consensus are used to determine K at which there is appreciable increase. The bar plot in (C) represents the mean consensus score for different numbers of clusters (K ranges from two to seven) on the basis of 100 repeated re-samplings of 80% of the participants.

**
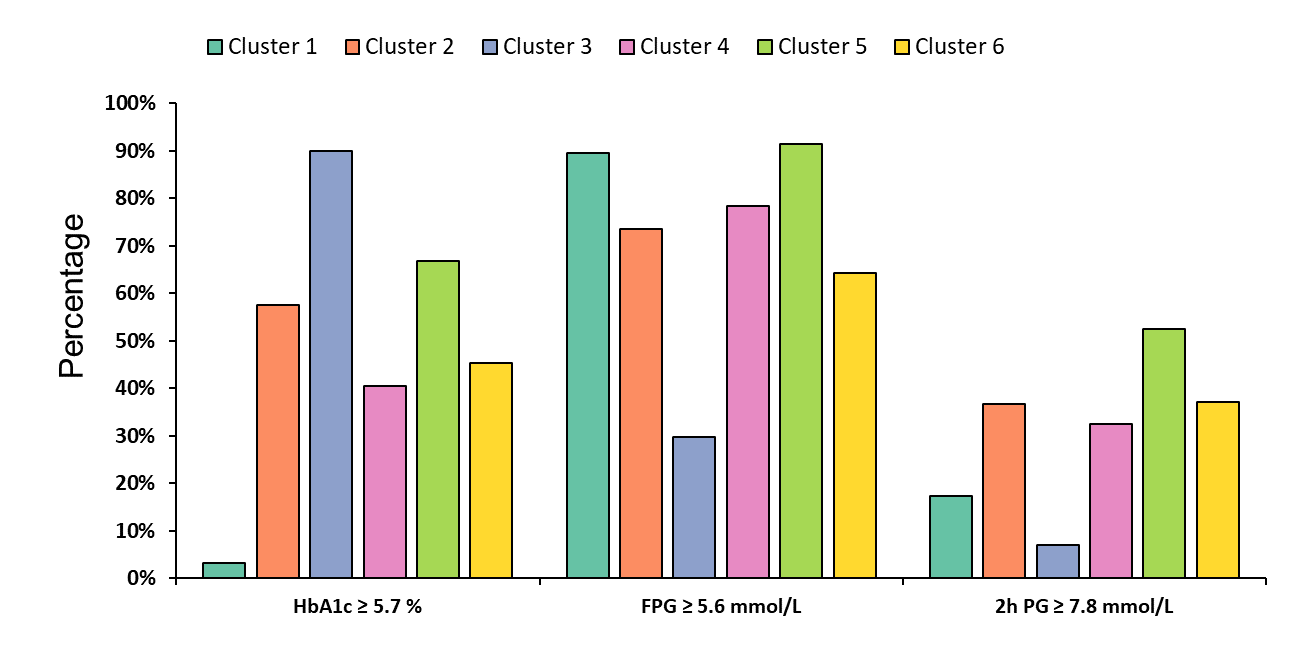
**

**Supplementary figure 4.** The percentages of participants in each cluster by glycemic categories


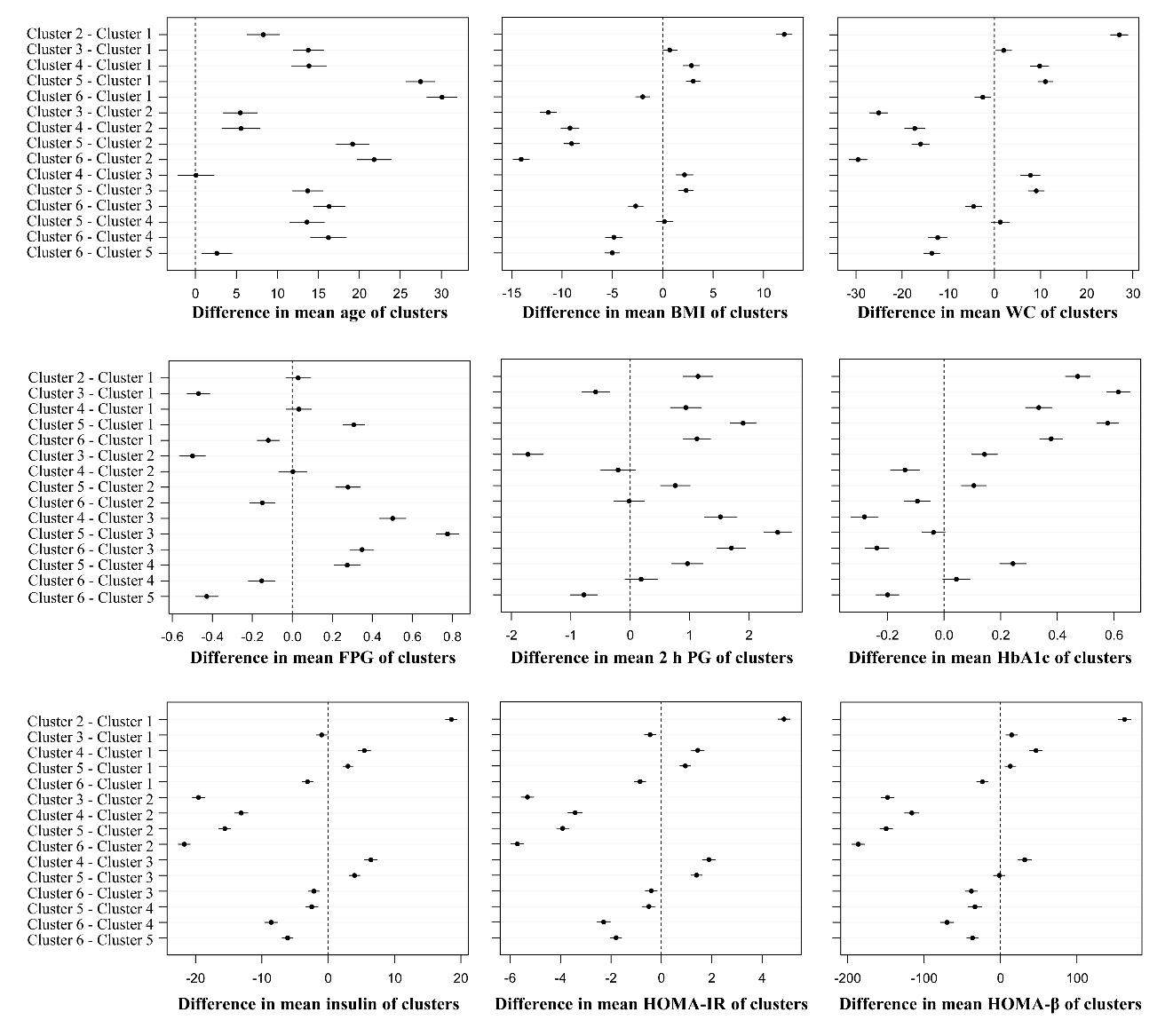


**Supplementary figure 5. The comparisons of the metabolic related factors between clusters by using Tukey test.**

The points and 95% confidence intervals denote the differences between two clusters, the more far from the zero line, the larger differences between two clusters. BMI, body mass index; WC, waist circumference; FPG, fasting plasma glucose; PG, postprandial glucose; HbA1c, glycated hemoglobin; HOMA-IR, homeostasis model assessment - insulin resistance; HOMA-β, homeostasis model assessment - β


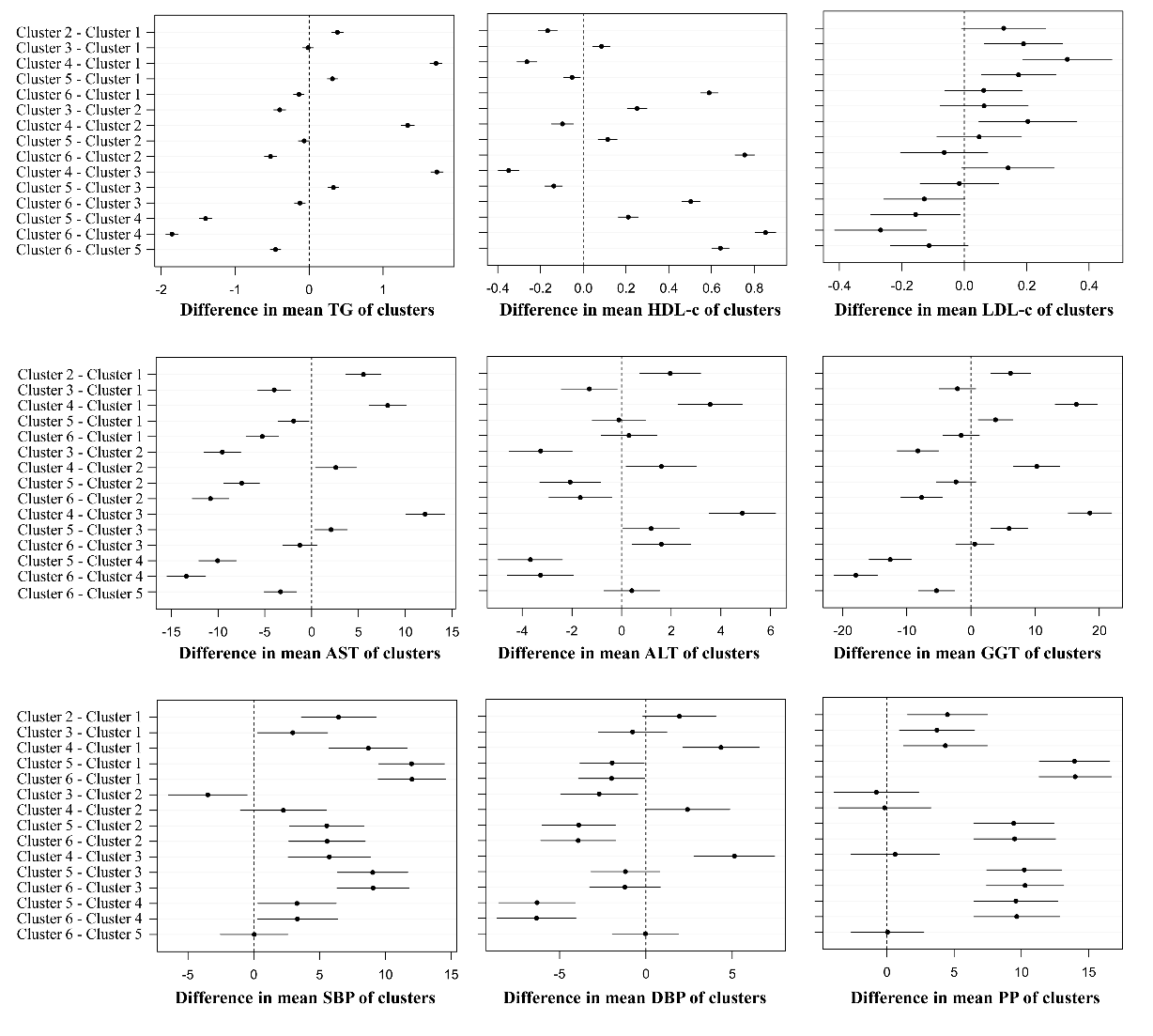


**Supplementary figure 6. The comparisons of the metabolic related factors between clusters by using Tukey test.**

The points and 95% confidence intervals denote the differences between two clusters, the more far from the zero line, the larger differences between two clusters. TG, triglyceride; HDL-c, high-density lipoprotein cholesterol; LDL-c, low-density lipoprotein cholesterol; AST, aspartate transaminase; ALT, alanine transaminase; GGT, glutamyl-transpeptidase; SBP, systolic blood pressure; DBP, diastolic blood pressure; PP, pulse pressure

**Supplementary table 1. Equations expressed for specified sex and serum creatinine level**

| Gender | Serum creatinine | Equation for estimating GFR |
| --- | --- | --- |
| Female | ≤0.7 mg/dl | 144 ◊ (SCr/0.7)^-0.329^ ◊ 0.993 ^Age^ [◊ 1.159 if black] |
| Female | >0.7 mg/dl | 144 ◊ (SCr/0.7)^-1.209^ ◊ 0.993 ^Age^ [◊ 1.159 if black] |
| Male | ≤0.9 mg/dl | 144 ◊ (SCr/0.9)^-0.411^ ◊ 0.993 ^Age^ [◊ 1.159 if black] |
| Male | >0.9 mg/dl | 144 ◊ (SCr/0.9)^-1.209^ ◊ 0.993 ^Age^ [◊ 1.159 if black] |

**Supplementary table 2**. The comparisons of cardiometabolic related factors between clusters

| Comparisons |  | Age |  |  |  | BMI |  |  |  | WC |  |
| --- | --- | --- | --- | --- | --- | --- | --- | --- | --- | --- | --- |
|  | Differences | SE | *P* value |  | Differences | SE | *P* value |  | Differences | SE | *P* value |
| Cluster 2 vs. Cluster 1 | -27.474 | 0.615 | < 0.001 |  | -3.021 | 0.236 | <0.001 |  | -11.052 | 0.575 | <0.001 |
| Cluster 3 vs. Cluster 1 | 2.618 | 0.642 | < 0.001 |  | -5.012 | 0.247 | <0.001 |  | -13.563 | 0.603 | <0.001 |
| Cluster 4 vs. Cluster 1 | -13.699 | 0.648 | < 0.001 |  | -2.327 | 0.249 | <0.001 |  | -9.062 | 0.607 | <0.001 |
| Cluster 5 vs. Cluster 1 | -19.187 | 0.701 | < 0.001 |  | 9.044 | 0.269 | <0.001 |  | 16.000 | 0.658 | <0.001 |
| Cluster 6 vs. Cluster 1 | -13.615 | 0.740 | < 0.001 |  | -0.167 | 0.285 | 0.992 |  | -1.263 | 0.692 | 0.446 |
| Cluster 3 vs. Cluster 2 | 30.091 | 0.639 | < 0.001 |  | -1.991 | 0.246 | <0.001 |  | -2.511 | 0.598 | <0.001 |
| Cluster 4 vs. Cluster 2 | 13.775 | 0.646 | < 0.001 |  | 0.694 | 0.248 | 0.058 |  | 1.990 | 0.602 | 0.012 |
| Cluster 5 vs. Cluster 2 | 8.287 | 0.698 | < 0.001 |  | 12.065 | 0.268 | <0.001 |  | 27.053 | 0.654 | <0.001 |
| Cluster 6 vs. Cluster 2 | 13.859 | 0.738 | < 0.001 |  | 2.855 | 0.284 | <0.001 |  | 9.789 | 0.688 | <0.001 |
| Cluster 4 vs. Cluster 3 | -16.317 | 0.672 | < 0.001 |  | 2.685 | 0.258 | <0.001 |  | 4.501 | 0.628 | <0.001 |
| Cluster 5 vs. Cluster 3 | -21.805 | 0.722 | < 0.001 |  | 14.056 | 0.278 | <0.001 |  | 29.563 | 0.678 | <0.001 |
| Cluster 6 vs. Cluster 3 | -16.232 | 0.761 | < 0.001 |  | 4.846 | 0.293 | <0.001 |  | 12.300 | 0.711 | <0.001 |
| Cluster 5 vs. Cluster 4 | -5.488 | 0.728 | < 0.001 |  | 11.371 | 0.280 | <0.001 |  | 25.063 | 0.682 | <0.001 |
| Cluster 6 vs. Cluster 4 | 0.084 | 0.766 | 0.999 |  | 2.160 | 0.295 | <0.001 |  | 7.799 | 0.714 | <0.001 |
| Cluster 6 vs. Cluster 5 | 5.572 | 0.811 | < 0.001 |  | -9.211 | 0.312 | <0.001 |  | -17.263 | 0.758 | <0.001 |

BMI, body mass index; WC, waist circumference; SE, standard error

**Continuous supplementary table 2**. The comparisons of cardiometabolic related factors between clusters

| Comparisons |  | FPG |  |  |  | 2 h PG |  |  |  | HbA1c |  |
| --- | --- | --- | --- | --- | --- | --- | --- | --- | --- | --- | --- |
|  | Differences | SE | *P* value |  | Differences | SE | *P* value |  | Differences | SE | *P* value |
| Cluster 2 vs. Cluster 1 | -0.307 | 0.019 | <0.001 |  | -1.901 | 0.077 | <0.001 |  | -0.578 | 0.014 | <0.001 |
| Cluster 3 vs. Cluster 1 | -0.428 | 0.020 | <0.001 |  | -0.779 | 0.080 | <0.001 |  | -0.200 | 0.014 | <0.001 |
| Cluster 4 vs. Cluster 1 | -0.776 | 0.020 | <0.001 |  | -2.482 | 0.081 | <0.001 |  | 0.037 | 0.014 | 0.09 |
| Cluster 5 vs. Cluster 1 | -0.278 | 0.021 | <0.001 |  | -0.761 | 0.087 | <0.001 |  | -0.106 | 0.015 | <0.001 |
| Cluster 6 vs. Cluster 1 | -0.275 | 0.023 | <0.001 |  | -0.964 | 0.092 | <0.001 |  | -0.243 | 0.016 | <0.001 |
| Cluster 3 vs. Cluster 2 | -0.120 | 0.019 | <0.001 |  | 1.123 | 0.080 | <0.001 |  | 0.378 | 0.014 | <0.001 |
| Cluster 4 vs. Cluster 2 | -0.469 | 0.020 | <0.001 |  | -0.581 | 0.080 | <0.001 |  | 0.615 | 0.014 | <0.001 |
| Cluster 5 vs. Cluster 2 | 0.029 | 0.021 | 0.745 |  | 1.141 | 0.087 | <0.001 |  | 0.472 | 0.015 | <0.001 |
| Cluster 6 vs. Cluster 2 | 0.032 | 0.022 | 0.700 |  | 0.937 | 0.092 | <0.001 |  | 0.334 | 0.016 | <0.001 |
| Cluster 4 vs. Cluster 3 | -0.349 | 0.020 | <0.001 |  | -1.704 | 0.084 | <0.001 |  | 0.237 | 0.015 | <0.001 |
| Cluster 5 vs. Cluster 3 | 0.149 | 0.022 | <0.001 |  | 0.018 | 0.090 | 0.991 |  | 0.094 | 0.016 | <0.001 |
| Cluster 6 vs. Cluster 3 | 0.153 | 0.023 | <0.001 |  | -0.185 | 0.095 | 0.368 |  | -0.044 | 0.017 | 0.091 |
| Cluster 5 vs. Cluster 4 | 0.498 | 0.022 | <0.001 |  | 1.722 | 0.091 | <0.001 |  | -0.143 | 0.016 | <0.001 |
| Cluster 6 vs. Cluster 4 | 0.502 | 0.023 | <0.001 |  | 1.519 | 0.096 | <0.001 |  | -0.281 | 0.017 | <0.001 |
| Cluster 6 vs. Cluster 5 | 0.003 | 0.025 | 0.998 |  | -0.203 | 0.101 | 0.334 |  | -0.138 | 0.018 | <0.001 |

FPG, fasting plasma glucose; 2 h PG, 2 hour postprandial glucose; SE, standard error

**Continuous supplementary table 2**. The comparisons of cardiometabolic related factors between clusters

| Comparisons |  | Insulin |  |  | HOMA-IR | | |  | HOMA-β | | |
| --- | --- | --- | --- | --- | --- | --- | --- | --- | --- | --- | --- |
|  | Differences | SE | *P* value |  | Differences | SE | *P* value |  | Differences | SE | *P* value |
| Cluster 2 vs. Cluster 1 | -2.991 | 0.268 | <0.001 |  | -0.954 | 0.074 | <0.001 |  | -12.938 | 2.484 | <0.001 |
| Cluster 3 vs. Cluster 1 | -6.115 | 0.280 | <0.001 |  | -1.797 | 0.077 | <0.001 |  | -36.517 | 2.595 | <0.001 |
| Cluster 4 vs. Cluster 1 | -3.965 | 0.283 | <0.001 |  | -1.398 | 0.078 | <0.001 |  | 1.587 | 2.620 | 0.991 |
| Cluster 5 vs. Cluster 1 | 15.579 | 0.306 | <0.001 |  | 3.915 | 0.084 | <0.001 |  | 149.314 | 2.831 | <0.001 |
| Cluster 6 vs. Cluster 1 | 2.471 | 0.323 | <0.001 |  | 0.493 | 0.089 | <0.001 |  | 33.368 | 2.991 | <0.001 |
| Cluster 3 vs. Cluster 2 | -3.124 | 0.279 | <0.001 |  | -0.844 | 0.077 | <0.001 |  | -23.579 | 2.584 | <0.001 |
| Cluster 4 vs. Cluster 2 | -0.974 | 0.282 | 0.007 |  | -0.444 | 0.077 | <0.001 |  | 14.525 | 2.609 | <0.001 |
| Cluster 5 vs. Cluster 2 | 18.570 | 0.304 | <0.001 |  | 4.869 | 0.084 | <0.001 |  | 162.252 | 2.821 | <0.001 |
| Cluster 6 vs. Cluster 2 | 5.462 | 0.322 | <0.001 |  | 1.447 | 0.088 | <0.001 |  | 46.305 | 2.981 | <0.001 |
| Cluster 4 vs. Cluster 3 | 2.150 | 0.293 | <0.001 |  | 0.399 | 0.081 | <0.001 |  | 38.103 | 2.715 | <0.001 |
| Cluster 5 vs. Cluster 3 | 21.694 | 0.315 | <0.001 |  | 5.713 | 0.087 | <0.001 |  | 185.831 | 2.919 | <0.001 |
| Cluster 6 vs. Cluster 3 | 8.586 | 0.332 | <0.001 |  | 2.290 | 0.091 | <0.001 |  | 69.884 | 3.074 | <0.001 |
| Cluster 5 vs. Cluster 4 | 19.544 | 0.318 | <0.001 |  | 5.313 | 0.087 | <0.001 |  | 147.727 | 2.942 | <0.001 |
| Cluster 6 vs. Cluster 4 | 6.436 | 0.334 | <0.001 |  | 1.891 | 0.092 | <0.001 |  | 31.781 | 3.096 | <0.001 |
| Cluster 6 vs. Cluster 5 | -13.108 | 0.354 | <0.001 |  | -3.422 | 0.097 | <0.001 |  | -115.946 | 3.276 | <0.001 |

HOMA-IR, homeostasis model assessment - insulin resistance; HOMA-β, homeostasis model assessment-β; SE, standard error

**Continuous supplementary table 2**. The comparisons of cardiometabolic related factors between clusters

| Comparisons |  | TG |  |  | HDL-c | | |  | LDL-c | | |
| --- | --- | --- | --- | --- | --- | --- | --- | --- | --- | --- | --- |
|  | Differences | SE | *P* value |  | Differences | SE | *P* value |  | Differences | SE | *P* value |
| Cluster 2 vs. Cluster 1 | -0.316 | 0.024 | <0.001 |  | 0.053 | 0.014 | 0.001 |  | -0.174 | 0.042 | <0.001 |
| Cluster 3 vs. Cluster 1 | -0.454 | 0.025 | <0.001 |  | 0.642 | 0.014 | <0.001 |  | -0.112 | 0.044 | 0.104 |
| Cluster 4 vs. Cluster 1 | -0.330 | 0.025 | <0.001 |  | 0.138 | 0.014 | <0.001 |  | 0.016 | 0.044 | 0.999 |
| Cluster 5 vs. Cluster 1 | 0.068 | 0.027 | 0.118 |  | -0.114 | 0.015 | <0.001 |  | -0.048 | 0.048 | 0.915 |
| Cluster 6 vs. Cluster 1 | 1.400 | 0.028 | <0.001 |  | -0.211 | 0.016 | <0.001 |  | 0.156 | 0.050 | 0.024 |
| Cluster 3 vs. Cluster 2 | -0.138 | 0.024 | <0.001 |  | 0.590 | 0.014 | <0.001 |  | 0.062 | 0.043 | 0.708 |
| Cluster 4 vs. Cluster 2 | -0.014 | 0.025 | 0.994 |  | 0.086 | 0.014 | <0.001 |  | 0.190 | 0.044 | <0.001 |
| Cluster 5 vs. Cluster 2 | 0.384 | 0.027 | <0.001 |  | -0.166 | 0.015 | <0.001 |  | 0.126 | 0.047 | 0.083 |
| Cluster 6 vs. Cluster 2 | 1.716 | 0.028 | <0.001 |  | -0.263 | 0.016 | <0.001 |  | 0.330 | 0.050 | <0.001 |
| Cluster 4 vs. Cluster 3 | 0.124 | 0.026 | <0.001 |  | -0.504 | 0.015 | <0.001 |  | 0.128 | 0.046 | 0.057 |
| Cluster 5 vs. Cluster 3 | 0.521 | 0.028 | <0.001 |  | -0.756 | 0.016 | <0.001 |  | 0.064 | 0.049 | 0.779 |
| Cluster 6 vs. Cluster 3 | 1.854 | 0.029 | <0.001 |  | -0.853 | 0.017 | <0.001 |  | 0.268 | 0.052 | <0.001 |
| Cluster 5 vs. Cluster 4 | 0.397 | 0.028 | <0.001 |  | -0.252 | 0.016 | <0.001 |  | -0.064 | 0.049 | 0.791 |
| Cluster 6 vs. Cluster 4 | 1.730 | 0.029 | <0.001 |  | -0.349 | 0.017 | <0.001 |  | 0.140 | 0.052 | 0.075 |
| Cluster 6 vs. Cluster 5 | 1.332 | 0.031 | <0.001 |  | -0.097 | 0.018 | <0.001 |  | 0.204 | 0.055 | 0.003 |

TG, triglyceride; HDL-c, high density lipoprotein cholesterol; LDL-c, low density lipoprotein cholesterol; SE, standard error

**Continuous supplementary table 2**. The comparisons of cardiometabolic related factors between clusters

| Comparisons |  | ALT |  |  | AST | | |  | GGT | | |
| --- | --- | --- | --- | --- | --- | --- | --- | --- | --- | --- | --- |
|  | Differences | SE | *P* value |  | Differences | SE | *P* value |  | Differences | SE | *P* value |
| Cluster 2 vs. Cluster 1 | 1.934 | 0.581 | 0.011 |  | 0.109 | 0.377 | 0.999 |  | -3.837 | 0.953 | <0.001 |
| Cluster 3 vs. Cluster 1 | -3.337 | 0.607 | <0.001 |  | 0.410 | 0.393 | 0.903 |  | -5.361 | 0.996 | <0.001 |
| Cluster 4 vs. Cluster 1 | -2.071 | 0.612 | 0.009 |  | -1.191 | 0.397 | 0.032 |  | -5.953 | 1.006 | <0.001 |
| Cluster 5 vs. Cluster 1 | 7.473 | 0.662 | <0.001 |  | 2.070 | 0.429 | <0.001 |  | 2.329 | 1.087 | 0.263 |
| Cluster 6 vs. Cluster 1 | 10.060 | 0.699 | <0.001 |  | 3.671 | 0.453 | <0.001 |  | 12.575 | 1.148 | <0.001 |
| Cluster 3 vs. Cluster 2 | -5.271 | 0.604 | <0.001 |  | 0.301 | 0.392 | 0.973 |  | -1.524 | 0.992 | 0.638 |
| Cluster 4 vs. Cluster 2 | -4.005 | 0.610 | <0.001 |  | -1.301 | 0.396 | 0.013 |  | -2.116 | 1.002 | 0.278 |
| Cluster 5 vs. Cluster 2 | 5.538 | 0.659 | <0.001 |  | 1.961 | 0.428 | <0.001 |  | 6.165 | 1.083 | <0.001 |
| Cluster 6 vs. Cluster 2 | 8.125 | 0.697 | <0.001 |  | 3.562 | 0.452 | <0.001 |  | 16.412 | 1.144 | <0.001 |
| Cluster 4 vs. Cluster 3 | 1.266 | 0.635 | 0.343 |  | -1.601 | 0.412 | 0.001 |  | -0.592 | 1.042 | 0.993 |
| Cluster 5 vs. Cluster 3 | 10.810 | 0.682 | <0.001 |  | 1.660 | 0.443 | 0.002 |  | 7.690 | 1.121 | <0.001 |
| Cluster 6 vs. Cluster 3 | 13.397 | 0.719 | <0.001 |  | 3.262 | 0.466 | <0.001 |  | 17.936 | 1.180 | <0.001 |
| Cluster 5 vs. Cluster 4 | 9.544 | 0.688 | <0.001 |  | 3.261 | 0.446 | <0.001 |  | 8.281 | 1.129 | <0.001 |
| Cluster 6 vs. Cluster 4 | 12.131 | 0.724 | <0.001 |  | 4.863 | 0.469 | <0.001 |  | 18.528 | 1.188 | <0.001 |
| Cluster 6 vs. Cluster 5 | 2.587 | 0.766 | 0.009 |  | 1.602 | 0.497 | 0.016 |  | 10.247 | 1.258 | <0.001 |

ALT, alanine transaminase; AST, aspartate transaminase; GGT, glutamyl-transpeptidase; SE, standard error

**Continuous supplementary table 2**. The comparisons of cardiometabolic related factors between clusters

| Comparisons |  | SBP |  |  | DBP | | |  | PP | | |
| --- | --- | --- | --- | --- | --- | --- | --- | --- | --- | --- | --- |
|  | Differences | SE | *P* value |  | Differences | SE | *P* value |  | Differences | SE | *P* value |
| Cluster 2 vs. Cluster 1 | -11.998 | 0.874 | <0.001 |  | 1.956 | 0.652 | 0.032 |  | -13.954 | 0.916 | <0.001 |
| Cluster 3 vs. Cluster 1 | 0.031 | 0.903 | 0.999 |  | -0.029 | 0.674 | 0.999 |  | 0.059 | 0.946 | 0.999 |
| Cluster 4 vs. Cluster 1 | -9.038 | 0.936 | <0.001 |  | 1.185 | 0.699 | 0.532 |  | -10.223 | 0.981 | <0.001 |
| Cluster 5 vs. Cluster 1 | -5.547 | 0.997 | <0.001 |  | 3.895 | 0.744 | <0.001 |  | -9.443 | 1.045 | <0.001 |
| Cluster 6 vs. Cluster 1 | -3.293 | 1.046 | 0.021 |  | 6.307 | 0.781 | <0.001 |  | -9.600 | 1.097 | <0.001 |
| Cluster 3 vs. Cluster 2 | 12.028 | 0.900 | <0.001 |  | -1.985 | 0.672 | 0.037 |  | 14.013 | 0.943 | <0.001 |
| Cluster 4 vs. Cluster 2 | 2.960 | 0.934 | 0.019 |  | -0.771 | 0.697 | 0.878 |  | 3.731 | 0.979 | 0.002 |
| Cluster 5 vs. Cluster 2 | 6.450 | 0.994 | <0.001 |  | 1.939 | 0.742 | 0.093 |  | 4.511 | 1.042 | <0.001 |
| Cluster 6 vs. Cluster 2 | 8.705 | 1.044 | <0.001 |  | 4.351 | 0.779 | <0.001 |  | 4.354 | 1.094 | <0.001 |
| Cluster 4 vs. Cluster 3 | -9.069 | 0.961 | <0.001 |  | 1.214 | 0.717 | 0.534 |  | -10.282 | 1.007 | <0.001 |
| Cluster 5 vs. Cluster 3 | -5.578 | 1.020 | <0.001 |  | 3.924 | 0.761 | <0.001 |  | -9.502 | 1.069 | <0.001 |
| Cluster 6 vs. Cluster 3 | -3.323 | 1.068 | 0.023 |  | 6.336 | 0.797 | <0.001 |  | -9.659 | 1.120 | <0.001 |
| Cluster 5 vs. Cluster 4 | 3.491 | 1.050 | 0.011 |  | 2.710 | 0.783 | 0.007 |  | 0.780 | 1.100 | 0.980 |
| Cluster 6 vs. Cluster 4 | 5.745 | 1.097 | <0.001 |  | 5.122 | 0.819 | <0.001 |  | 0.623 | 1.150 | 0.994 |
| Cluster 6 vs. Cluster 5 | 2.255 | 1.149 | 0.362 |  | 2.412 | 0.857 | 0.055 |  | -0.157 | 1.204 | 0.999 |

SBP, systolic blood pressure; DBP, diastolic blood pressure; PP, pulse pressure; SE, standard error

**Supplementary table 3.** The association between prediabetes clusters with hypertension defined as systolic blood pressure ≥ 140 mmHg or diastolic blood pressure ≥ 90 mmHg or currently taking antihypertensive medicine

|  | Cluster 1 | Cluster 2 | Cluster 3 | Cluster 4 | Cluster 5 | Cluster 6 |
| --- | --- | --- | --- | --- | --- | --- |
| High systolic blood pressure or taking antihypertensive medicine |  |  |  |  |  |  |
| Prevalence, % | 0.15 (0.11 - 0.18) | 0.45 (0.39 - 0.51) | 0.32 (0.26 - 0.37) | 0.42 (0.35 - 0.48) | 0.59 (0.54 - 0.65) | 0.49 (0.44 - 0.53) |
| Unadjusted OR | Reference | 4.74 (3.38 – 6.63) | 2.67 (1.84 – 3.88) | 4.12 (2.88 - 5.91) | 8.47 (6.32 - 11.32) | 5.48 (3.97 - 7.58) |
| Adjusted OR * | Reference | 2.36 (1.36 - 4.08) | 1.47 (0.77 - 2.77) | 4.12 (2.14 - 7.93) | 5.52 (3.26 - 9.35) | 5.93 (3.46 - 10.17) |
| High diastolic blood pressure or taking antihypertensive medicine |  |  |  |  |  |  |
| Prevalence, % | 0.13 (0.10 - 0.16) | 0.42 (0.36 - 0.47) | 0.28 (0.23 - 0.33) | 0.37 (0.30 - 0.44) | 0.51 (0.45 - 0.56) | 0.39 (0.35 - 0.44) |
| Unadjusted OR | Reference | 4.79 (3.34 - 6.86) | 2.66 (1.81 - 3.92) | 3.96 (2.63 - 5.94) | 6.92 (4.97 – 9.66) | 4.36 (3.01 - 6.31) |
| Adjusted OR * | Reference | 2.91 (1.13 - 7.54) | 0.75 (0.29 - 1.96) | 6.82 (2.42 - 19.20) | 2.50 (0.75 - 8.34) | 1.92 (0.59 - 6.29) |
| Hypertension |  |  |  |  |  |  |
| Prevalence | 0.16 (0.13 - 0.19) | 0.46 (0.40 - 0.52) | 0.32 (0.26 - 0.37) | 0.44 (0.37 - 0.51) | 0.60 (0.54 - 0.65) | 0.49 (0.44 - 0.54) |
| Unadjusted OR | Reference | 4.49 (3.20 - 6.30) | 2.46 (1.70 - 3.54) | 4.17 (2.88 - 6.04) | 7.89 (5.86 - 10.62) | 5.03 (3.67 - 6.90) |
| Adjusted OR * | Reference | 2.35 (1.32 - 4.16) | 1.26 (0.66 - 2.39) | 4.47 (2.27 - 8.80) | 4.80 (2.85 - 8.09) | 4.76 (2.87 - 7.90) |

Data are weighted prevalence rate (95% confidence interval) and weighted odds ratio (95% confidence interval)

High systolic blood pressure was defined systolic blood pressure ≥ 140 mmHg; high diastolic blood pressure was defined as diastolic blood pressure ≥ 90 mmHg.

* Adjusted model was adjusted for gender, smoking status, drinking status, education level, physical activity, taking antihypertensive medicine, taking lipid lowering medicine.
